# A Healthier Smile in the Past? Dental Caries and Diet in Early Neolithic Farming Communities from Central Germany

**DOI:** 10.3390/nu14091831

**Published:** 2022-04-27

**Authors:** Nicole Nicklisch, Vicky M. Oelze, Oliver Schierz, Harald Meller, Kurt W. Alt

**Affiliations:** 1 Center for Natural and Cultural History of Man, Faculty of Medicine/Dentistry, Danube Private University, Förthofstraße 2, 3500 Krems, Austria; kurt.alt@dp-uni.ac.at; 2 Anthropology Department, University of California at Santa Cruz, 1156 High Street, Santa Cruz, CA 96064, USA; voelze@ucsc.edu; 3 Department of Prosthodontics and Materials Science, University of Leipzig, Liebig Str. 12, 04103 Leipzig, Germany; oliver.schierz@medizin.uni-leipzig.de; 4 State Office for Heritage Management and Archaeology Saxony-Anhalt and State Museum of Prehistory, Richard-Wagner Str. 9, 06114 Halle, Germany; hmeller@lda.stk.sachsen-anhalt.de; 5 Institute of Prehistory and Archaeological Science, Department of Environmental Sciences, University of Basel, Spalenring 145, 4055 Basel, Switzerland

**Keywords:** caries, nutrition, oral health, stable isotope analysis, bioarcheology

## Abstract

Dental health is closely linked to an individual’s health and diet. This bioarcheological study presents dental caries and stable isotope data obtained from prehistoric individuals (*n* = 101) from three Early Neolithic sites (c. 5500-4800 BCE) in central Germany. Dental caries and ante-mortem tooth loss (AMTL) were recorded and related to life history traits such as biological sex and age at death. Further, we correlate evidence on caries to carbon and nitrogen isotope data obtained from 83 individuals to assess the relationship between diet and caries. In 68.3% of the adults, carious lesions were present, with 10.3% of teeth affected. If AMTL is considered, the values increase by about 3%. The prevalence of subadults (18.4%) was significantly lower, with 1.8% carious teeth. The number of carious teeth correlated significantly with age but not sex. The isotopic data indicated an omnivorous terrestrial diet composed of domestic plants and animal derived protein but did not correlate with the prevalence of carious lesions. The combined evidence from caries and isotope analysis suggests a prevalence of starchy foods such as cereals in the diet of these early farmers, which aligns well with observations from other Early Neolithic sites but contrasts to Late Neolithic and Early Bronze Age populations in Germany.

## 1. Introduction

In modern-day societies, caries is the most widespread non-communicable disease [1]. The nature of dental caries and its association with diet and oral hygiene are well understood [2,3]. Once the homeostasis of the physiological oral microbiome is disturbed, this may favour the colonization and multiplication of pathogenic bacteria [4,5]. *Streptococcus mutans* is the main representative of caries-promoting pathogens, which have the ability to metabolize low-molecular-weight carbohydrates such as sugars quite rapidly, producing organic acids such as lactic acid. An excess of acid-producing bacteria leads to the demineralization of tooth enamel, which can ultimately result in failure of the overall tooth structure and tooth loss. The bacterial infection can even spread to the jawbone and lead to so-called periapical alterations or abscesses, thus causing further complications [6]. Dental caries is a multifactorial disease in which individual dietary habits (food, processing, texture), oral hygiene, genetic factors (e.g., enamel thickness, microstructure, tooth morphology, saliva composition), and underlying pathological conditions may also play a role [2,4,7]. As dental health has an impact on a physiological and socio-cultural level, age- and sex-specific associations with caries have been described [8,9].

In the past, a sharp increase in dental caries correlated significantly with fundamental changes in human subsistence strategies and diet [9]. The first important event was the adoption of agricultural practices to procure food in the Neolithic period. Particularly the cultivation of several species of cereal provided a readily available and, thus, secure source of food. The higher starch and, hence, carbohydrate content in this novel diet and the way food was processed resulted in a higher prevalence of caries [9,10]. With the availability of cane and beet sugars in the 18th and 19th centuries, caries occurrence and frequency increased significantly [10]. However, in many industrialized Western nations, dental health has improved in the past few decades, especially among children and adolescents. The main elements are new concepts for prevention and treatment and the desire for wellbeing [3]. Nevertheless, the incidence of caries is still much higher in Western industrialized nations than in most African and Asian populations [11].

In archeological and forensic contexts, teeth can still provide information even when other tissue structures have already been destroyed by taphonomic processes [12,13]. Analyses of pathophysiological changes in dental hard tissue provide information on the frequency and severity of dental disease within a population, as well as information on individual and population-specific behaviors and dietary habits [9,14,15]. In addition, it is possible to visualize spatial and chronological trends in oral health in human history through comparative analysis of dental pathology data [8,10,16,17].

Our study provides an insight into the dental health of the earliest farmers of central Europe, who had their genetic origins in the Near East and settled in central Germany approximately 7000 years ago [18,19]. In addition to a (1) detailed survey of dental caries and ante-mortem tooth loss (AMTL) in three burial communities, this study puts the occurrence of dental caries in relation to stable isotope data obtained from human bone collagen, assessing the possible relationship between diet and dental disease [20]. Stable isotope analysis of carbon and nitrogen can provide information on the main source of dietary proteins and carbohydrates and allows us to differentiate between herbivore, omnivore, carnivore, and piscivore diets [21,22]. We expect to find (2) lower frequencies of dental caries in individuals with slightly higher nitrogen isotope values and, hence, higher amounts of animal protein (milk, meat) in their diet [20]. Another question is (3) how biological sex and (4) age relate to the prevalence of dental caries and AMTL in these populations. Gender-based differences in diet have been described for several Neolithic sites in Europe, with male individuals tending to show isotopic evidence of more frequent animal protein consumption than female individuals [23].

## 2. Materials and Methods

### 2.1. Material

**Figure 1:**
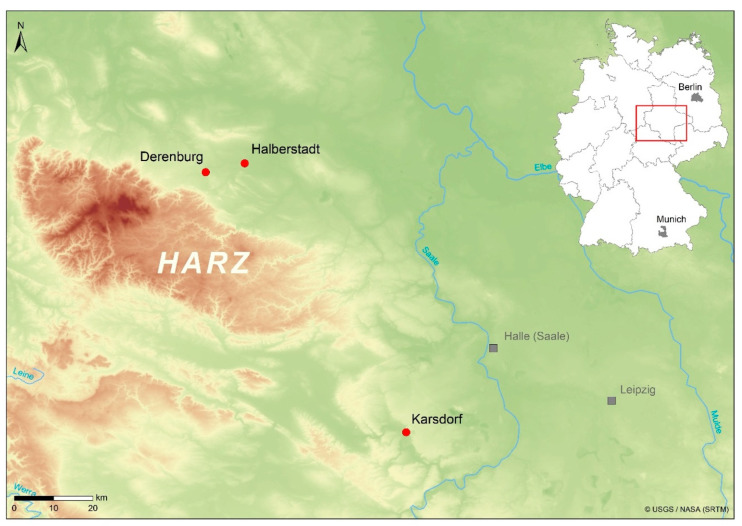
Location of the three sites in the Middle Elbe-Saale region, Saxony-Anhalt, central Germany (LDA, Halle/Saale).

This bioarcheological study includes data from the three Early Neolithic sites of Halberstadt-Sonntagsfeld, Derenburg-Meerenstieg II, and Karsdorf-Steigra in the Middle Elbe-Saale region (MES) of central Germany (Figure 1). Based on its characteristic ceramic ornamentation, the archaeological culture is referred to as linear pottery culture (Linearbandkeramik = LBK) [24]. The MES is bordered in the west and south by low mountain ranges (Harz, Thuringian Forest, and Ore Mountains) and in the east by glacial landscapes. The area is interspersed with fertile loess soils, which offered favorable conditions for settling and farming in prehistoric times [25,26]. In the western regions of the MES in particular, the lime-rich soils support the preservation of bones. The human remains sampled in this study were radiocarbon-dated to the period between 5450 and 4775 cal BC [27].

Two of the sites, Derenburg-Meerenstieg II (DEB) and Halberstadt-Sonntagsfeld (HBS), are located in the foothills of the northern Harz region (Nordharzvorland, Harz district, Saxony-Anhalt), at a distance of less than 10 km from each other. With its 47 burials, DEB can be defined as a cemetery. In contrast, the 41 skeletons from HBS were found in so-called settlement burials, i.e., grouped together close to the remains of wooden longhouse structures. Single burials dominated at HBS and DEB, but a few double burials were also found. More than half of the burials contained grave goods such as pottery, earth pigments, and spondylus shells [28]. The third site, Karsdorf-Steigra (KAR) in the Unstrut Valley (Burgenland), is located approximately 100 km from the other sites. As at HBS, the burials at KAR were also found within the settlement. The 30 individuals were grouped primarily to the west of the longhouse structures. All deceased were buried in individual grave pits, and the number of grave goods can be described as low compared to the other sites [29].

### 2.2. Methods

#### 2.2.1. Age and Sex Determination

Following international recommendations, a broad range of morphometric methods were used for age and sex determination of the skeletal remains [30,31]. The age determination in adult individuals (>20 years) was based on the assessment of the cranial suture closure [32], changes in the auricular surface [33,34], and the pubic symphysis [35]. In children and adolescents (<20 years = subadults), age determination was based on the tooth development and eruption [36], long-bone lengths [37], and ossification patterns of epiphyses and apophyses [38]. The sex was determined on the basis of morphological and metric criteria in adults only [38,39,40]. Age determinations were made according to the following classification [41]: *infans* I (0–6 years), *infans* II (7–14 years), *juvenis* (15–20 years), *adultas* (21–40 years), *maturitas* (41–60 years), *senilis* (≥61 years).

#### 2.2.2. Morphological Examination of the Teeth

Assessment of the dental status was based on the designation standards of the Fédération Dentaire Internationale (FDI) [42]. Adapted to the bioarcheological evaluation of dental caries, the following two quotients were defined: (a) caries frequency (or prevalence) in percent (CF = number of affected individuals × 100/number of assessable individuals), (b) caries experience in percent (CE = number of affected teeth × 100/number of assessed teeth) [43,44,45]. Carious lesions (dental cavities) were divided into five stages: *caries superficialis* (1), *caries media* (2), *caries profunda* without *pulpa aperta* (3), *caries profunda* with *pulpa aperta* (4), *radix relicta* (5) [12]. So-called “white spots” and “brown spots” were not part of this evaluation.

DMF scores are not appropriate for archeological investigations, because teeth may be missing due to periodontal disease, trauma, or heavy tooth wear, and teeth lost post-mortem (PMTL) may also have been carious [43,46]. Nevertheless, in the present study ante-mortem tooth loss (AMTL) was assessed and combined with carious lesions in relation to the assessable alveolar sockets (dental alveoli). AMTL was considered if the alveolus was completely remodeled or at least showed signs of remodeling. The number of assessable dental alveoli is composed of the number of preserved teeth, AMTL, and PMTL.

#### 2.2.3. Digital Volume Tomography (DVT)

For a more detailed dental analysis of some individuals (*n* = 13), radiological images were taken at the Department of Prosthodontics and Materials Science, Leipzig University using digital volume tomography (DVT, Morita 3D Accuitomo 170), with a slice thickness of 1 mm and a voxel size of 0.250 (tube voltage: 80 kV; tube current: 2.0 mA).

#### 2.2.4. Stable Isotope Data

To assess whether the occurrence and prevalence of carious lesions can be associated with dietary patterns in the three study populations, stable carbon and nitrogen isotope data, δ^13^C and δ^15^N, respectively, previously published by Oelze and colleagues [20], were also taken into account. Depending on the state of preservation, the bones used for isotope analysis were mainly ribs, but a few long bones and skull fragments were also sampled. Stable isotopes in collagen samples were extracted following the procedure outlined by Richards and Hedges [47] and analyzed in a Flash EA 2112 coupled to a DeltaXP isotope ratio mass spectrometer (Thermo-Finnigan, Bremen, Germany) at the Max Planck Institute for Evolutionary Anthropology in Leipzig, Germany. The δ^13^C and δ^15^N values are reported here in ‰ following the international standards vPDB and AIR. The analytical error was better than 0.2‰ (1σ) for both isotope systems [20]. Collagen quality was affirmed by inspecting %nitrogen, %carbon, and the atomic C:N ratios for each sample following the recommendations by Ambrose [48].

#### 2.2.5. Statistical Data Analysis

To compare the prevalence of caries with life history parameters and stable isotope data in this archeological population with post-mortem tooth loss, we calculated percent caries (caries%) as the number of teeth affected by caries divided by the number of teeth present per individual. This allows us to assess dental caries in light of age, sex, site, and dietary patterns across individuals. We ran two linear regression models in R (version R 4.1.1, [49]) with the alpha level set to 0.05. One model was run using the full dataset (*n* = 83), thus testing the effect of age (average value in years), site (3 levels: DEB, HBS, KAR), and δ^13^C and δ^15^N values on the percentage of teeth affected by caries per individual (%caries/individual). Log1p transformations were conducted to remove the zeros from the response variable %caries/individual in both models. The fixed effect of sex was also included in the second model, having excluded all subadult individuals and those of undetermined sex from the dataset (*n* = 64). In the full dataset model, the possible interaction between δ^13^C and δ^15^N was initially tested; this, however, was not significant (χ^2^ = 0.0, df = 76, *p* = 0.958) and was subsequently dropped from the full model. The interaction between isotopes was not considered in the second model.

Model diagnostics were carried out on both models by visually inspecting histograms, qq-plots, and residuals plotted against fitted values, all of which confirmed normally distributed and homogeneous residuals. Testing variance inflation factors in both models found no evidence for collinearity (vifs around 1–1.2). The final results were obtained by comparing both full models with a null model each, which only contained the fixed effect of site, using chi-square independence tests.

## 3. Results

Out of a total of 116 skeletons recovered from the three Neolithic sites, the dentitions of 101 individuals could be examined for this study (Table 1). These comprised 63 adults and 38 subadults (<20 years) with 1910 permanent and 277 deciduous teeth (Appendix A Table A1). For a total of 83 individuals, we can report both dental caries and isotope analysis results.

**Figure 2:**
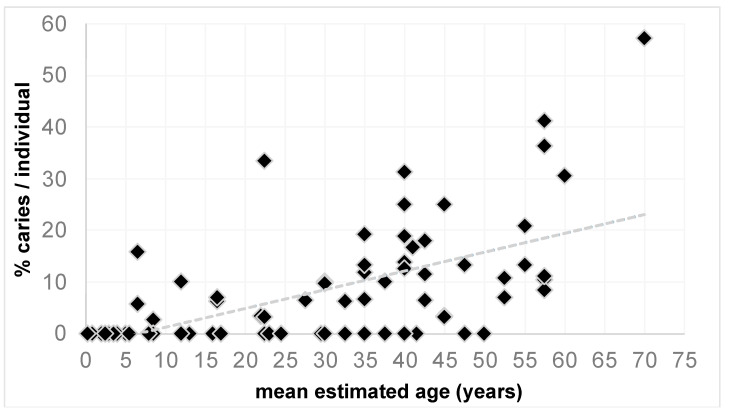
Scatterplot showing the significant relationship between the mean estimates of age in years and the percentage of caries per individual.

The model testing the effects of age, site, and δ^13^C and δ^15^N values in relation to the percentage of carious teeth per individual (*n* = 83) was highly significant (χ^2^ = 65.4, df = 77, *p* < 0.000). However, as shown in Figure 2, this was driven exclusively by the effect of age (*p* < 0.000). Interestingly, none of the other predictors had any impact on the percentage of caries per individual (site: *p*= 0. 878, δ^13^C: *p* = 0.594, δ^15^N: *p* = 0.433). A similar pattern emerged from the second model which included the fixed effect of sex in a subset of all adult individuals. Here too the full model was highly significant (χ^2^ = 43.2, df = 57, *p* < 0.000), exclusively driven by the effect of age (*p* < 0.000) and not by site (*p* = 0.613), sex (*p* = 0.733) or δ^13^C (*p* = 0.669), and δ^15^N (*p* = 0.431) values.

### 3.1. Age-Specific Differences

Table 2 summarizes the caries frequencies (CF) and caries experience (CE) for all adult and subadult individuals in this study. At DEB, 18 out of the 28 adults (64.3%) were affected by caries. The CF in the adults from HBS (12/17, 70.6%) and KAR (13/18, 72.2%) were slightly higher, but the number of assessable individuals was lower. A CF of 68.3% (43/63) can be determined for all three groups taken together. When considering CE, the adults from HBS exhibited the highest number of carious teeth (12.0%), followed by the group from KAR, with a CE of 10.9%. The adult group from DEB showed the lowest CE rate of 8.6%. Combining all three LBK sites, a CE value of 10.3% can be determined. In most cases, a small number of individuals were characterized by particularly severe dental cavities. AMTL could only be detected in adult individuals. Of 63 adults, 14 are affected (22.2%). Individuals from KAR are most frequently affected by AMTL; in relation to the assessable dental alveoli, HBS is in the lead. In total, two adults show AMTL but no carious lesions, which raises the combined frequency (AMTL + caries) by individuals to 71.4%. Looking at the combined data in relation to the assessable alveolar sockets, the average frequency increases by 3%.

Among the subadults, the CF value was highest at KAR with 30% (Table 2). At DEB, two out of 12 (16.7%) and at HBS two out of 16 subadults (12.5%) were affected. Overall, seven of 38 subadult individuals (18.4%) had carious lesions. With 4–5 affected teeth, the CE values in the subadults of all three sites were low. At KAR, 2.9% of the preserved teeth were affected and at DEB, 1.6%. The lowest CE rate (1.4%) can be determined for the subadult group from HBS. Combining the data of the subadult individuals from all three LBK sites results in a CE rate of 1.8%. AMTL was not observed among the subadult group.

A closer look at all adult individuals by age group (Table 3) suggests an increase in both CF and CE with advancing age. The same tendency can be described for AMTL and AMTL combined with caries, both at the individual level and by alveolar sockets. However, the oldest age group (>61) is only represented by a single individual.

The subadults affected are exclusively children of the *infans* II age group (7–14 years) and juvenile individuals. Carious deciduous teeth were observed in the *infans* II group. Carious lesions are detectable in the permanent molars of an approximately 12-year-old child and two juvenile individuals (15–20 years). No carious lesions were found in the *infans* I group (0–6 years).

### 3.2. Sex-Related Differences

The results of our statistical data analysis show no significant effect of sex on the percentage of carious teeth per individual among all adults from the three Neolithic sites. However, although the sample sizes per archeological site are small, we can describe some trends in our dataset. At DEB and KAR, females (81.3% and 83.3%) tend to be more frequently affected by dental caries than males (40.0% and 63.6%) (Table 4). At HBS, the situation is different: all six male individuals show evidence of dental cavities, but only six out of 11 females (54.5%) have carious lesions. Overall, CF appears slightly higher for females (72.7%) than for males (63.0%). At the individual level AMTL is slightly higher in females, but the combined data from AMTL and caries do not lead to any significant changes.

Similar results occur when looking at the CE values. Again, males from DEB and KAR (5.9% and 4.9%, respectively) seem less affected by carious teeth than the females (10.2% and 21.6%). At HBS, males (13.7%) tend to show more carious lesions than females (11.5%). The female individuals from KAR show the highest CE with 21.6%. AMTL is more common in females than in males. In comparison, the combined data from AMTL and caries show a small but non-significant increase in frequencies.

### 3.3. Affected Tooth Types

The adult individuals from all three sites are summarized in Table 5. The distribution shows a clear trend, whereby the post-canine teeth are clearly more affected by carious lesions than the anterior teeth. The first molar (tooth 16; 22.4%) and the second premolar (tooth 15; 20.4%) are most affected in the right maxilla. In the left maxilla, the most dental cavities are found on the second molar (tooth 27; 33.3%), followed by the first molar (tooth 26; 22.9%). In the right and left mandibles, most defects are evenly distributed among the molars, with third molars being slightly more affected.

Due to the low prevalence in the subadults, a tabular presentation was omitted. A total of 13 teeth, eight deciduous teeth and five permanent teeth, are affected. The affected deciduous teeth are seven molars and one canine. The affected permanent teeth are four second molars and one first molar.

### 3.4. Severity of Caries Lesions

When looking at the adult individuals from all sites, most teeth affected by caries show only minor lesions in the form of *caries superficialis* (grade 1) and *caries media* (grade 2) (Table 6). These two forms of caries represent approximately 60% of all defects. Deeper structural defects, such as *caries profunda* without (grade 3) or with opened pulp cavity (grade 4), are less frequently observed considering the total number of affected teeth. There are differences between the sites: at DEB, the proportion of grade 4 cavities (25%) is higher, whereas, at KAR, the proportion of grade 3 (19.2%) and grade 5 (21.2%) is higher. HBS also shows a higher proportion of grade 5 defects (20.4%), which are associated with the complete destruction of the dental crowns.

In the subadult individuals, the severity does not exceed grade 3. Of the eight deciduous teeth, two show grade 3, four teeth show grade 2, and two show grade 1. Of the five permanent teeth, three exhibit grade 1, one shows grade 2, and two show grade 3.

### 3.5. Stable Isotope Ratios and Diet

The stable isotope ratios for carbon and nitrogen for the three study populations have been reported and discussed previously by Oelze and colleagues [20]. No significant differences in stable isotope values were found between the sites. The stable isotope values in all fully weaned individuals (estimated age older than 5 years) cluster tightly in δ^13^C with an average value of −19.8‰ (±0.3‰ 1σ). The δ^15^N values for these individuals show slightly more variation with an average value of 8.8‰ (±0.7‰ 1σ). Subadults tend to have higher δ^13^C and even higher δ^15^N values than adult individuals.

## 4. Discussion

This paper presents the dental caries profiles and matching stable isotope records of three early Neolithic farming populations to describe the nuanced relationship between diet and dental heath in one of the earliest agricultural societies of central Europe. The comparison between HBS, DEB, and KAR is problematic due to the small number of individuals per burial community. Our statistic model did not pick up any differences between sites in the percentage of caries per individual, and the three sites were also found to be almost indistinguishable isotopically [20]. This suggests that, in respect of their dental health, we can treat all individuals sampled as one study population. The marginal differences between sites, such as the lower CF and CE of the adult individuals from DEB or the higher caries burden in the males from HBS, are of limited significance. The combined data of AMTL and caries show a relatively small, non-significant increase in the frequencies of age- and sex-specific differences. Overall, the three LBK populations show a rather moderate degree of dental cavities in which the milder forms of caries such as caries superficialis and caries media dominate. The results can be discussed under various aspects, with nutrition playing an essential role.

### 4.1. The Influence of Nutrition

The δ^13^C data point to a dependence on domesticated C3-plants for these early farmers, whereas the possibility of prevalent wild plant foods and C4-plant cultivation can be excluded [20,50,51]. The δ^15^N values suggest that this domestic plant-based diet was low in leguminous plants which fix nitrogen directly from the soil [21] and was mixed with considerable amounts of animal-derived protein, most likely meat from domestic cattle, sheep, goats, and pigs [20]. A previous comparison of human δ^15^N values with those of domestic animals from each site suggested that individuals from DEB may have had slightly less animal protein in their everyday diet than the inhabitants of the two other sites [20]. If that is the case, we would predict that the plant and, hence, possibly cereal proportion of the diet might have been higher for DEB community members. However, this assumption does not align with the CF and CE values of the DEB individuals, which are very similar to those of the other two sites.

Based on the genetic evidence from several individuals from DEB, we can say that these people were likely not lactase-persistent and, hence, could not digest lactose from unfermented milk after reaching maturity [52]. As expected, children younger than five years (*n* = 13) have slightly elevated δ^13^C and δ^15^N values compared to older individuals, as their isotope values are influenced by the tropic level increase associated with human breastmilk consumption [20,53]. Isotopic differences between adult males and females are insignificant and do not suggest that men had considerably better access to meat than women. Overall, the isotopic evidence indicates that all individuals led an agricultural lifestyle with a mixed diet of domesticated plants (cereals and small amounts of legumes) and animal products [20].

Cereals occupied an important position in the diet of Early Neolithic farming cultures. The geographic region studied offered rich loess soils and, thus, optimal conditions for cereal cultivation. Archeological investigations show that the LBK people preferred to settle in these loess areas [24,25]. The cultivation of cereals increased the proportion of carbohydrate-rich food. New food processing techniques made starch and sugar easily accessible to bacteria of the oral cavity, thus promoting the development of caries [10]. Studies indicate that early farmers suffered an increase in caries prevalence compared to hunter-gatherers [9]. In addition to diet, genetic factors also affect the quality of tooth structure and saliva production [2,4]. Nevertheless, an essential role in cariogenesis can be attributed to the starch and sugar content in the diet [2,54]. Epidemiological studies suggest an increased caries risk when starch is combined with sugar [55]. In the European Early Neolithic, fruits and honey can be assumed as sweet foods. Genetic analyses of dental calculus samples from different time periods demonstrate that the composition of the oral flora from the Neolithic to the Middle Ages was more diverse than that of recent populations [56]. This trend can be attributed to continuous changes in the human diet.

### 4.2. Age-Specific Differences and Caries Localisation

At 68.3%, the CF was significantly higher in the adults than in the subadult individuals, at 18.4%. The difference in CE was also very clear, where the proportion of affected teeth was 5.7 times higher in adults than in children and adolescents. In principle, young children between 0 and 6 years of age were not affected. Only in the older children and adolescents a few carious teeth were found, including some deciduous teeth. The nutritional data indicated that children older than three years were weaned. Before that, there does not seem to have been any risk of dental caries, e.g., by feeding premasticated food.

**Figure 3:**
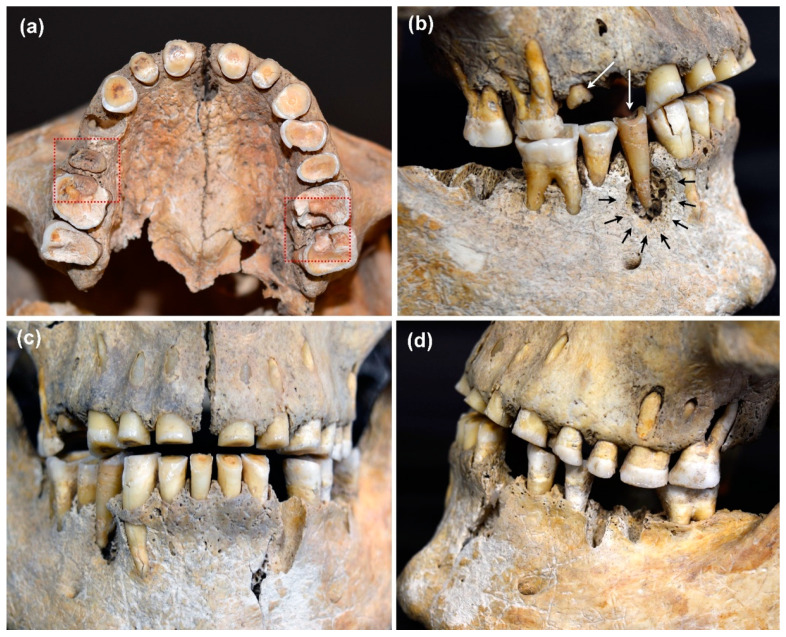
(**a**–**d**). Dentition of an adult male individual (ID 484) from DEB (**a**) In the upper molars (teeth 16, 26, 27), the carious lesions appear to originate in the interdental spaces (approximal caries; red rectangles). (**b**) In the two premolars (teeth 15, 44) in the right upper and lower jaw (white arrows), the crowns are destroyed, and the origin of the carious lesion cannot be reconstructed. The area around the first mandibular premolar (tooth 44) shows traces of an abscess (black arrows). (**c**) Dental wear is particularly pronounced in the anterior dentition. Incisors and canines of both maxilla and mandible show formation of secondary dentin (cf. (**a**)). (**d**) There is evidence of periapical changes on molars 26 and 27. Teeth 33, 36, and 47 were taken for DNA analyses, tooth 14 was lost post-mortem (DEB 484, 35–45 years, male).

**Figure 4:**
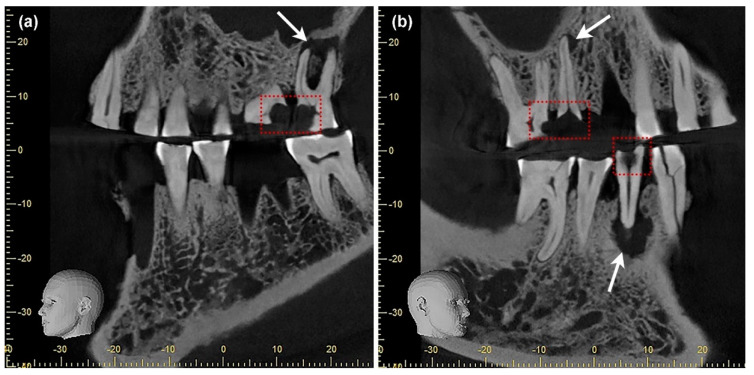
(**a**,**b**) The DVT images show that the dental cavities in some teeth (red rectangles) have led to periapical changes in the bone (white arrows): (**a**) in the left maxilla, the second molar (tooth 27) is affected; (**b**) in the right maxilla, there is a small lesion on the second premolar (tooth 15); and on the canine of the left mandible (tooth 33), there is a larger cavity that can be identified as a possible cyst due to its size. The latter has led to a vestibular abscess (cf. (**b**); DEB 484, 35–45 years, male).

The data confirm that the risk of dental caries increased significantly with age. Within the dentition, caries was almost exclusively found in the post-canine teeth. Few cavities in the anterior teeth were found in older group members who were severely affected by caries. The carious lesions seem to have originated predominantly on the approximal surfaces of the interdental space. An impressive example is the dentition of a 35–45-year-old male individual (ID 484) from DEB (Figure 3a–d). The morphological examination shows pronounced carious lesions on a total of five teeth, three maxillary molars (teeth 16, 26, 27), and two premolars (teeth 15, 44). In the three maxillary molars, the carious lesions originate on the approximal surfaces (Figure 3a). Severe wear is noticeable on the occlusal surfaces and is particularly pronounced in the anterior region (Figure 3a,c). Periapical changes resulting from deep caries lesions with opening of the pulp cavity are visible on teeth 26, 27, and 44 (Figure 3a,d). These periapical lesions can be clearly localized in DVT images of the maxilla and mandible (Figure 4a,b). Due to the osteolytic processes in the jaw bones, it is very likely that the man suffered from recurrent inflammatory reactions. Without medical intervention, chronic oral infections can spread to the adjacent tissue and the entire body [57,58,59,60].

### 4.3. Sex-Related Differences

In the present study, females had a slightly higher CF (72.7%) than males (63.0%). The difference was even more pronounced for CE. Although this difference is not statistically significant, female individuals had more carious teeth, with a CE of 13.1%, than male individuals with 7.5%. The same trend can be observed considering AMTL.

Differences between the sexes are not uncommon in archeological and ethnological contexts, and a higher carious burden is often reported in females [9,61,62]. This is usually explained by a difference in diet, with females consuming less animal protein than males. It is supposed that females consume more carbohydrate-rich foods to meet their primary caloric needs [16,63,64]. However, a higher proportion of animal protein in the diet has a more beneficial effect on dental health [10]. The proteins found in dairy products, especially casein, reduce caries activity by inhibiting bacterial attachment to enamel as well as decreasing the solubility of hydroxyapatite, thus counteracting demineralization [65,66,67]. However, it cannot be ruled out that women preferred foods with a higher starch or sugar content, such as porridge, fruits, and honey.

In addition to dietary habits, hormonal differences between the sexes can also play a role [63,68]. For example, females show lower salivary flow rates than males [69,70]. Furthermore, high estrogen levels during pregnancy have a negative effect on the quantity and composition of saliva [71,72]. This favors the colonization of cariogenic bacteria and the development of periodontal diseases, which indirectly affects tooth loss. This explains the observed higher number of AMTL in females in the present study and the correlation between births and tooth loss in other studies [73,74,75].

### 4.4. Influence of Dental Wear

Occlusal wear protects against occlusal caries to a certain extent by preventing plaque from adhering to the tooth [10]. Mastication also promotes wear at the contact points of the teeth, which is called interproximal (interstitial) wear. The continuous progression of both occlusal and interstitial wear reduces the contact surfaces between the teeth [76]. As a result, the interdental spaces are better flushed with saliva, which protects the teeth from approximal caries (e.g., anterior teeth Figure 3a). However, if the wear continues, there is a high probability that the pulp cavity will be exposed. In the past, this could lead to the death and subsequent loss of the tooth [6]. Compared to current industrialized nations, dental wear was well developed in Neolithic populations, as shown in Figure 3c,d. The main reason for this was mechanical abrasion induced by dietary habits and can be explained by the higher proportion of rough, fiber-rich food as well as naturally occurring sand and grit in processed food [10,77,78]. Flour is a perfect example, as it was much more coarsely ground in ancient times and contained grit due to the use of grinding stones [79]. A total of 74 adult individuals from the sites investigated were subjected to an analysis of the degree of dental wear [80]. The results showed that the degree of wear of the occlusal surfaces continuously increased with age and reached its maximum level in mature and senile individuals. A comparison between the anterior and posterior regions showed that the mean degree of dental wear was higher in anterior teeth than in post-canines. Slightly more wear was observed in the upper jaw, especially in the anterior teeth. Differences between the upper and lower jaw were attributed to a higher load on the upper teeth due to occupational activities and the use of teeth as a third hand [81,82].

### 4.5. Comparative Data from Other Neolithic Sites

Comparing the prevalence of caries in different populations from the same or from neighboring geographic regions can provide information about the living conditions and subsistence strategies (Table 7). Comparative data from early farming populations (LBK/BK) from central Germany show a similar range of CE values for adults [83,84], with a considerably higher CE at Wandersleben [85]. If AMTL is included in the calculations, the frequencies increase and align with those from Wandersleben and Sondershausen. The low variability within the CE values does not indicate any significant nutritional differences within early farming populations in the MES. Data from the LBK site at Aiterhofen (southern Bavaria) show a similar CE value for adults, but the CF differs significantly [86]. In contrast, a lower CE was observed in adults from the Early Neolithic site of Kleinhadersdorf in Lower Austria [87]. In the subadults, the CE ranged between 1 and 3%; at Aiterhofen [88] and Kleinhadersdorf [87] the proportion of affected deciduous teeth was higher. The CF showed a greater range of variation in both adults and subadults. Different environmental conditions and subsistence strategies must be expected in different geographic regions. Isotope analyses from southern Germany, for example, show higher δ^15^N values (mean: 9.7‰) than in the MES, which is interpreted as evidence of a higher proportion of animal protein in the diet [89]. In addition, sample size and differences in the age structure of the investigated populations may also contribute to the variations.

Lower CF and CE values were observed for the later periods of the Neolithic (MN, LN) and for the Early Bronze Age (EBA) [17,90,91]. This indicates a change in subsistence and dietary patterns. The results of C/N analyses from the Middle and Late Neolithic and the Early Bronze Age suggest that the amount of animal protein in the diet increased [92]. The higher proportion of meat and dairy products might have had a positive effect not only on dental health but also on the general wellbeing. The diachronic comparison suggests that the early farmers’ diet was characterized by a higher proportion of carbohydrates, which can be attributed primarily to the cultivation of cereals [17,25].

## 5. Conclusions

The aim of this bioarcheological study was to provide insights into the relationship of dental caries and diet in c. 7000-year-old Early Neolithic farmers from central Germany. The results show that, although consuming a diet lower in sugar content compared to modern populations, carious lesions were not uncommon among early farmers. We found no significant differences between the sexes or between burial communities but, as anticipated, a strong effect of age on the presence of dental caries. A diet rich in carbohydrates can be blamed for this, with grain consumption playing an important part. Single cases illustrate the painful fate of some older individuals. It is evident that caries acquired symptomatic significance with increasing age, while carious lesions did not yet play a role in infants still relying strongly on mother’s milk. The absence of a sex effect is supportive of the notion of a comparatively egalitarian lifestyle, in which both sexes have equivalent access to plant and animal-based foods.

## Figures and Tables

**Table 1:** Archeological sites, number (N) of excavated individuals separated in adults and subadults, and number of individuals with preserved teeth separated in adults and subadults.

Archeological Sites	N Excavated Individuals	N Adults	N Subadults	N Individuals with Teeth	N Adults with Teeth	N Subadults with Teeth
DEB	47	32	15	40	28	12
HBS	38	18	20	33	17	16
KAR	31	20	11	28	18	10
Total	116	70	46	101	63	38

**Table 2:** Caries frequency (CF) and caries experience (CE) in adult and subadult individuals for each site and in total. Information on AMTL and AMTL combined with caries is given by adult individuals and assessable alveolar sockets.

Site	Adults		Subadults	
	Individuals	CF %	Individuals	CF %
DEB	18/28	64.3	2/12	16.7
HBS	12/17	70.6	2/16	12.5
KAR	13/18	72.2	3/10	30.0
total	43/63	68.3	7/38	18.4
	Teeth	CE %	Teeth	CE %
DEB	52/594	8.8	4/255	1.6
HBS	49/409	12.0	4/281	1.4
KAR	52/477	10.9	5/171	2.9
total	153/1480	10.3	13/707	1.8
	**Adults with AMTL**			
	AMTL by individuals	%	AMTL by alveolar sockets	%
DEB	5/28	17.9	9/625	1.4
HBS	3/17	17.6	23/453	5.1
KAR	6/18	33.3	23/517	4.4
total	14/63	22.2	55/1595	3.4
	AMTL + caries by individuals	%	AMTL + caries by alveolar sockets	%
DEB	19/28	67.9	61/625	9.8
HBS	12/17	70.6	72/453	15.9
KAR	14/18	77.8	75/517	14.5
total	45/63	71.4	208/1595	13.0

**Table 3:** Caries frequency (CF) and caries experience (CE) in subadult and adult individuals from all sites by age group, with data on permanent teeth and deciduous teeth affected. Data on AMTL and AMTL combined with caries are given by adult individuals and assessable alveolar sockets.

Age (Years)	Indidviduals	%	Permanent Teeth/Alveolar Sockets	%	Deciduous Teeth	%
	Caries (CF)		Caries by teeth (CE)			
0–6	0/19	0.0	0/105	0.0	0/188	0.0
7–14	5/13	38.5	2/194	1.0	8/87	9.2
15–20	2/6	33.3	3/131	2.3	0/2	0.0
21–40	22/37	59.5	75/873	8.6	---	---
41–60	20/25	80.0	66/586	11.3	---	---
>61	1/1	100	12/21	57.1	---	---
	AMTL		AMTL by alveolar sockets			
21–40	3/37	8.1	13/917	1.4	---	---
41–60	10/25	40.0	37/652	5.7	---	---
>61	1/1	100	5/26	19.2	---	---
all adults	14/63	22.2	55/1595	3.4	---	---
	AMTL + caries		AMTL + caries by alveolar sockets			
21–40	22/37	59.5	88/917	9.6	---	---
41–60	22/25	84.0	103/652	15.8	---	---
>61	1/1	100	17/26	65.4	---	---
all adults	45/63	71.4	208/1595	13.0	---	---

**Table 4:** Sex-specific differences in caries frequency (CF), caries experience (CE). Data on AMTL and AMTL combined with caries are given by individuals and assessable alveolar sockets.

Site	Male	%	Female	%	Male	%	Female	%
	Caries by individuals (CF)				Caries by teeth (CE)			
DEB	4/10	40.0	13/16	81.3	13/221	5.9	38/365	10.4
HBS	6/6	100	6/11	54.5	24/175	13.7	27/234	11.5
KAR	7/11	63.6	5/6	83.3	14/285	4.9	35/162	21.6
Total	17/27	63.0	24/33	72.7	51/682	7.5	100/761	13.1
	AMTL by individuals				AMTL by alveolar sockets			
DEB	0/10	0.0	5/16	31.2	0/224	0.0	9/393	2.3
HBS	1/6	16.7	2/11	18.2	6/185	3.2	17/268	6.3
KAR	3/11	27.3	3/6	50.0	12/313	3.8	11/174	6.3
Total	4/27	14.8	10/33	30.3	18/722	2.5	37/835	4.4
	AMTL + caries by individuals				AMTL + caries by alveolar sockets			
DEB	4/10	40.0	14/16	87.5	13/224	5.8	47/393	12.0
HBS	6/6	100	6/11	54.5	28/185	15.1	44/268	16.4
KAR	8/11	72.7	5/6	83.3	26/313	8.3	46/174	26.4
Total	18/27	66.7	25/33	75.7	67/722	9.3	137/835	16.4

**Table 5:** Distribution of carious lesions in the dentition of adult individuals from all sites with information on the number of assessable (N teeth) and carious teeth (N affected). The teeth were named according to the FDI system.

	Right Jaw								Left Jaw							
Upper Jaw	18	17	16	15	14	13	12	11	21	22	23	24	25	26	27	28
N teeth	31	49	49	49	49	51	44	42	45	47	47	47	51	48	48	27
N affected	4	7	11	10	8	4	2	1	1	1	2	2	6	11	16	5
% affected	12.9	14.3	22.4	20.4	16.3	7.8	4.5	2.4	2.2	2.1	4.3	4.3	11.8	22.9	33.3	18.5
Lower Jaw	48	47	46	45	44	43	42	41	31	32	33	34	35	36	37	38
N teeth	35	50	49	51	48	51	47	45	40	47	49	51	53	49	51	40
N affected	6	7	7	5	2	1	0	1	0	1	1	1	4	9	9	8
% affected	17.1	14.0	14.3	9.8	4.2	2.0	0.0	2.2	0.0	2.1	2.0	2.0	7.5	18.4	17.6	20.0

**Table 6:** Degree of caries in adult individuals: number of affected teeth (N teeth) by severity of carious lesion (grade 1–5).

Grade	DEB		HBS		KAR		Total	
	N Teeth	%	N Teeth	%	N Teeth	%	N Teeth	%
1	11	21.2	20	40.8	19	36.5	50	32.7
2	24	46.2	9	18.4	9	17.3	42	27.5
3	3	5.8	7	14.3	10	19.2	20	13.1
4	13	25.0	3	6.1	3	5.8	19	12.4
5	1	1.9	10	20.4	11	21.2	22	14.4
Total	52	100	49	100	52	100	153	100

**Table 7:** Caries frequency (CF) and caries experience (CE) amongst adult and subadult individuals compared to other Neolithic and Early Bronze Age datasets from the MES region, Germany.

Period/Site	CF %		CE %		References
	Subadult	Adult	Subadult	Adult	
DEB	26.7	64.3/69.9 **	1.6	8.8/9.8 **	this study
HBS	12.5	70.6/70.6 **	1.4	12.0/15.9 **	this study
KAR	30.0	72.2/77.8 **	2.9	10.9/14.5 **	this study
All sites	18.4	68.3/71.4 **	1.2/2.9 *	10.3/13.0 **	this study
BK/Wandersleben (MES)	---	63.8	---	14.4	[85] **
BK/Wandersleben (MES)	12.5	---	3.2/0.0 *	---	[88]
BK/Sondershausen (MES)	---	69.0	---	11.8	[83] **
BK/collection	---	58.1	---	11.3	[84]
LBK/Aiterhofen (SB)	36.8	---	5.4/2.8 *	---	[88]
LBK/Aiterhofen (SB)	---	37.0	---	9.2	[86]
LBK/Kleinhadersdorf (LA)	---	60.7	4.9/2.0 *	7.3	[87]
MN/collection (MES)	8.0	44.0	1.3	4.9	[17]
LN/collection (MES)	9.8	38.3	0.9	5.5	[17]
LN/collection (MES)	---	36.4	---	6.0	[90] **
EBA/collection (MES)	11.4	35.6	0.9	5.8	[17]
EBA/collection (MES)	---	38.3	---	6.9	[91] **

* deciduous teeth/permanent teeth; ** ante-mortem tooth loss included; BK (Bandkeramik, c. 5700–4100 BC), MN (Middle Neolithic, 3950–3025 cal BC), LN (Late Neolithic, 2800–2050 cal BC), EBA (Early Bronze Age, 2200–1575 cal BC); SB (Southern Bavaria, Germany), LA (Lower Austria).

## Data Availability

All relevant data are contained in the article.

## Appendix A

**Table A1:** Number of individuals, permanent teeth, deciduous teeth and teeth lost ante-mortem (AMTL) of the three burial sites by age groups.

Age (Years)	Individuals	Permanent Teeth	Deciduous Teeth	AMTL
		**Derenburg**		
0–6	6	54	50	0
7–14	4	84	23	0
15–20	2	44	0	0
21–40	18	390	0	4
41–60	10	204	0	5
>61	0	0	0	0
total	40	776	73	9
**Halberstadt**				
0–6	8	40	83	0
7–14	5	65	33	0
15–20	3	58	2	0
21–40	10	231	0	9
41–60	7	178	0	14
>61	0	0	0	0
total	33	572	118	23
**Karsdorf**				
0–6	5	11	55	0
7–14	4	45	31	0
15–20	1	29	0	0
21–40	9	252	0	0
41–60	8	204	0	18
>61	1	21	0	5
total	28	562	86	23
